# Increased selenium and decreased iron levels in relation to risk of coronary artery disease in patients with diabetes

**DOI:** 10.3389/fnut.2023.1103330

**Published:** 2023-05-18

**Authors:** Mengyun Tian, Teng Hu, Jiajun Ying, Hanbin Cui, Ning Huangfu

**Affiliations:** ^1^Department of Cardiology, The First Affiliated Hospital of Ningbo University, School of Medicine, Ningbo University, Ningbo, China; ^2^Cardiovascular Disease Clinical Medical Research Center of Ningbo, Ningbo, China; ^3^Department of Cardiology, Ningbo First Hospital, Ningbo, China; ^4^Key Laboratory of Precision Medicine for Atherosclerotic Diseases of Zhejiang Province, Ningbo, China

**Keywords:** micronutrient, coronary artery disease, diabetes, causal association, selenium, iron

## Abstract

**Background:**

Observational studies have reported inconsistent associations between micronutrient levels and the risk of coronary artery disease (CAD) in diabetic patients. We aim to explore the causal association between genetically predicted concentrations of micronutrients (phosphorus, magnesium, selenium, iron, zinc, and copper) and CAD in patients with diabetes.

**Methods:**

Single nucleotide polymorphisms (SNPs) connected to serum micronutrient levels were extracted from the corresponding published genome-wide association studies (GWASs). Summary-level statistics for CAD in diabetic patients were obtained from a GWAS of 15,666 patients with diabetes. The primary analysis was carried out with the inverse variance weighted approach, and sensitivity analyses using other statistical methods were further employed to assess the robustness of the results.

**Results:**

Genetically predicted selenium level was causally associated with a higher risk of CAD in diabetic patients (odds ratio [OR]: 1.25; 95% confidence interval [CI]: 1.10–1.42; *p* = 5.01 × 10^−4^). While, genetically predicted iron concentrations in patients with diabetes were inversely associated with the risk of CAD (OR: 0.82; 95% CI: 0.75–0.90; *p* = 2.16 × 10^−5^). The association pattern kept robust in most sensitivity analyses. Nominally significant associations were observed for magnesium and copper with the risk of CAD in patients with diabetes. No consistent evidence was found for the causal associations between phosphorus and zinc levels, and the risk of CAD in patients with diabetes.

**Conclusion:**

We provide consistent evidence for the causal effect of increased selenium and decreased iron levels on CAD in patients with diabetes, highlighting the necessity of micronutrient monitoring and application in these patients.

## Introduction

Coronary artery disease (CAD) remains the leading cause of death worldwide, especially in patients with diabetes (1, 2). Since CAD is responsible for more than 50% of diabetes-related mortality, it dictates the prognosis for diabetic patients (3). Therefore, the 2019 European Society of Cardiology (ESC) guidelines have clarified the importance of preventing CAD in patients with diabetes (4).

Growing evidence from observational studies and randomized controlled trials (RCTs) indicated that essential micronutrients may play a critical role in the development of CAD in people with diabetes, but the results were inconsistent (5–7). For example, a meta-analysis including 40 prospective cohort studies with over 1 million individuals has shown that increasing dietary magnesium intake was associated with a reduced risk of diabetes and all-cause mortality, but not CAD or total cardiovascular diseases (CVDs) (8). Observational studies found the negative or no association between selenium biomarkers and CAD (9, 10), however, the RCTs revealed that decreased heart disease mortality among individuals with diabetes was related to increased selenium concentration (11). On the one hand, as observational studies based on reports of participants were subjected to confounding factors, which might be inaccurate leading to biased results (12). On the other hand, due to the limits of the sample size, the evidence from RCTs may not be powerful enough to evaluate the causal effect of micronutrients on the risk of CAD in diabetic patients (13).

Mendelian randomization (MR) approach can be applied to explore the potential causal association between exposure and disease by using genetic variants as instrumental variables (IVs) (14). The constraints of observational studies are successfully resolved by the random assignment of genotype at conception and the non-influence of genetic variations by potential confounding variables (15). In the current study, a two-sample MR analysis was conducted to investigate the causal associations between the risk of CAD in diabetic patients and circulating concentrations of six systematically selected micronutrients, including phosphorus, magnesium, selenium, iron, zinc, and copper.

## Methods

### Study design

**Figure 1:**
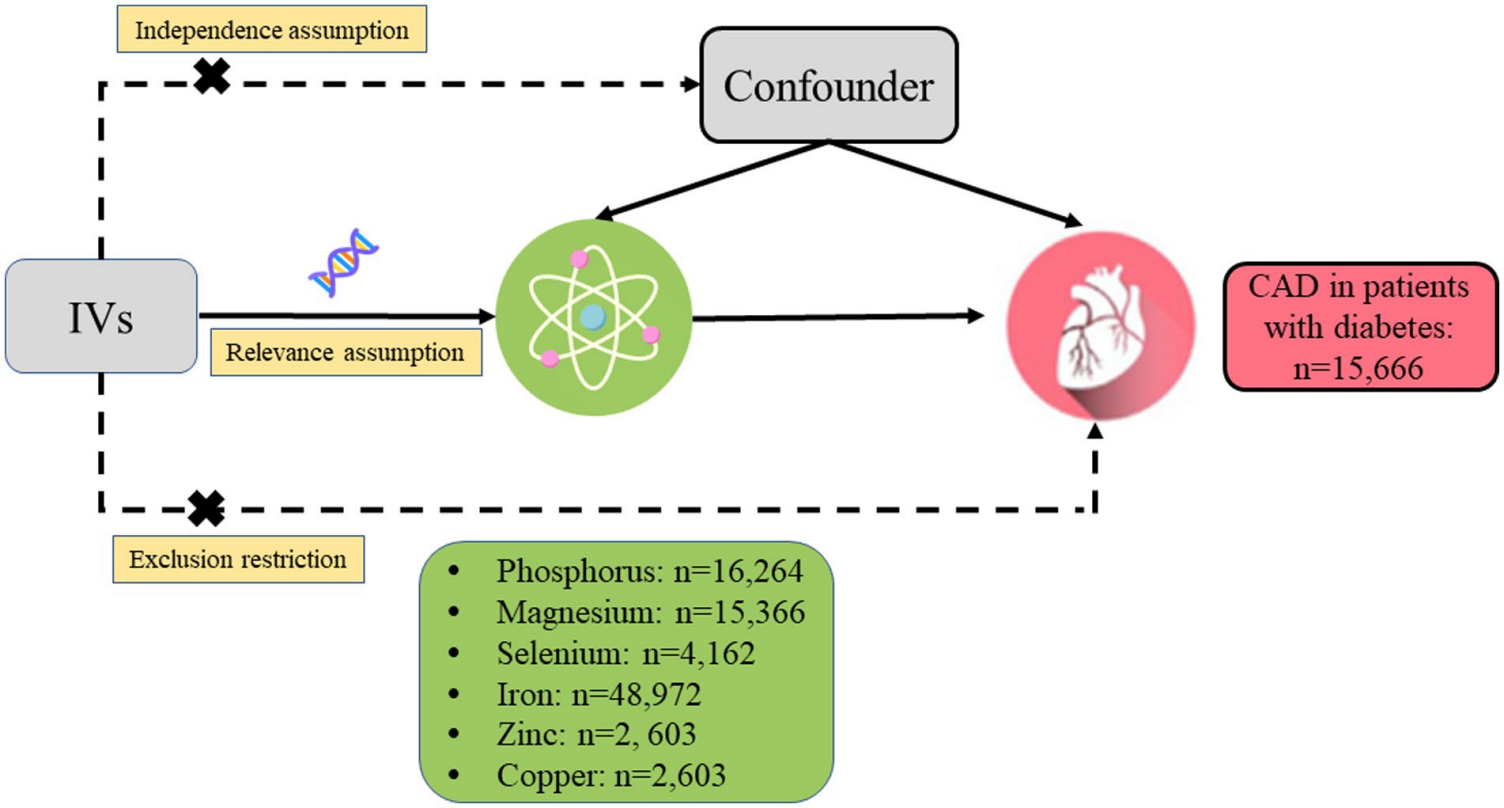
Design of the current two-sample Mendelian randomization study. Three core assumptions were as follows: (α) Relevance assumption; (β) Independence assumption; (γ) Exclusion restriction. IVs, instrumental variables; CAD, coronary artery disease.

A two-sample MR analysis was designed to estimate the causal relationship between genetically determined circulating micronutrient concentrations and the risk of CAD in diabetic patients (Figure 1). The following three core assumptions should be met by the single nucleotide polymorphisms (SNPs) chosen as IVs for circulating concentrations of micronutrients: (1) IVs should be closely related to the circulating concentrations of micronutrients, (2) IVs should be independent of any potential confounders, and (3) IVs should be associated with the risk of CAD in patients with diabetes only through the concentrations of micronutrients.

### Genetic instrument selection

First, SNPs were obtained from recently published genome-wide association studies (GWASs) that independently affect these nutrient concentrations at the genome-wide significance level (*p* < 5 × 10^−8^). Then, the linkage disequilibrium tests were performed based on the European 1000 Genomes Project reference panel (*r*^2^ < 0.01). If two SNPs were in linkage disequilibrium, the one with smaller value of *p* would be kept. Considering palindromic SNPs, those with minor allele frequency larger than 0.42 were regarded as not inferable and removed. Specifically, SNPs linked to serum phosphorus levels were extracted from a large GWAS meta-analysis including 16,264 participants of European ancestry (16). Six SNPs that achieved genome-wide significance in the joint analysis of the discovery (*n* = 15,366 participants) and replication (*n* = 8,463 participants) cohorts from European descent were utilized as genetic IVs for serum magnesium concentration (17). The GWAS meta-analysis of log-transformed toenail selenium concentrations and standardized residuals of log-transformed blood selenium concentrations, which included up to 4,162 individuals in four United States studies, provided the genetic summary data for serum selenium levels (18). The genetic association with serum iron levels was derived from the Genetics of Iron Status consortium, with up to 48,972 participants (19). The GWAS meta-analysis employing two cohorts from Australia and the United Kingdom yielded the SNPs chosen as genetic IVs for zinc and copper concentrations (20).

### Data for outcome

The summary statistics for CAD in patients with diabetes were extracted from the recently published GWAS, including 15,666 patients of European ancestry with diabetes (3,968 CAD cases and 11,696 controls) from the United Kingdom Biobank (21). The average age at diabetes diagnosis was 52.4 ± 12.2 for CAD cases (Male: 2,936; 74.0%) and it was 51.2 ± 12.6 for controls (Male: 7,037; 60.2%). The average age at visit was 62.7 ± 5.6 and 60.2 ± 7.0 for individuals with or without CAD, respectively.

All of the studies in our analyses have obtained relevant ethics review approvals, and all the participants included in the original studies provided written informed permission. All the data used in the current study had been publicly available.

### Statistical analysis

The multiplicative random-effects inverse-variance weighted (IVW) method was employed as the primary analysis to evaluate the effect of genetically predicted micronutrient concentrations on the risk of CAD in diabetic individuals. Specifically, the causal estimate for each SNP was generated using the Wald estimator, and the corresponding standard error was calculated using the Delta method. Subsequently, the overall estimate was calculated by meta-analyzing all the estimates by the IVW method (22).

To further validate the accuracy of the findings, the Maximum likelihood (22), Weighted median (23), MR-Egger regression (24), and Mendelian Randomization Pleiotropy Residual Sum and Outlier (MR-PRESSO) methods were applied in follow-up sensitivity analyses (25). For instance, the Maximum likelihood method could provide a greater empirical power of estimates as it assumed that the genetic association between risk factors and outcomes follows a bivariate normal distribution (22). The Weighted median method could still produce reliable estimates even if ≤50% of the weight comes from the ineffective SNPs (23). Intercept tests could be used in the MR-Egger regression to assess the potential horizontal pleiotropy (24). The MR-PRESSO method was conducted to identify potential outliers and, after eliminating them, to provide relatively unbiased causal estimates (25). In addition, scatter plots and leave-one-out analyses were performed to depict the associations of genetically determined micronutrient levels with CAD in patients with diabetes. However, sensitivity analyses and leave-one-out analyses could not be performed as the number of SNPs for zinc and copper was less than three. Cochran’s *Q* statistics and corresponding value of *p* were calculated to assess the degree of heterogeneity in the IVW analyses (26). Considering the Bonferroni adjustment for multiple tests, a value of *p* of <0.008 (0.05/6 exposures) was deemed statistically significant. The value of *p*s between 0.008 and 0.05 were considered to indicate suggested associations. All the statistical analyses were conducted by R Software (version 4.1.1.; R Foundation for Statistical Computing, Vienna, Austria), the R package TwoSampleMR,^1^ and MR-PRESSO.^2^

## Results

Two to seven SNPs genetically determining the serum phosphorus levels were identified as IVs for serum phosphorus, magnesium, selenium, iron, zinc, and copper levels, respectively (Table 1). In the MR analysis, all *F*-statistic values of the genetic tools were above the suggested threshold of 10 (Table 1).

**Table 1:** Characteristics of the single-nucleotide polymorphisms associated with serum micronutrients levels and coronary artery disease in patients with diabetes.

Exposure	SNP	Chr	Pos	EA	OA	EAF	*F*	Micronutrients			CAD in patients with diabetes		
								Beta	SE	*p* value	Beta	SE	*p* value
Phosphorus	rs1697421	1	21,823,292	C	T	0.49	100	0.050	0.005	1.14E−27	0.033	0.027	0.217
Phosphorus	rs17265703	3	122,048,644	G	A	0.85	36	0.036	0.006	4.32E−09	0.042	0.038	0.259
Phosphorus	rs9469578	6	33,706,479	T	C	0.92	43	0.059	0.009	1.11E−11	−0.021	0.052	0.684
Phosphorus	rs947583	6	136,133,659	T	C	0.29	49	0.035	0.005	3.45E−12	0.036	0.030	0.224
Phosphorus	rs2970818	12	4,606,168	T	A	0.09	35	0.047	0.008	4.38E−09	−0.029	0.044	0.510
Magnesium	rs11144134	9	77,499,796	C	T	0.08	121	0.011	0.001	8.20E−15	0.062	0.048	0.191
Magnesium	rs13146355	4	77,412,140	A	G	0.44	25	0.005	0.001	6.30E−13	0.033	0.027	0.220
Magnesium	rs3925584	11	30,760,335	T	C	0.55	36	0.006	0.001	5.20E−16	0.007	0.027	0.791
Magnesium	rs4072037	1	155,162,067	T	C	0.54	100	0.010	0.001	2.00E−36	0.012	0.027	0.640
Magnesium	rs448378	3	169,100,899	A	G	0.53	16	0.004	0.001	1.30E−08	0.019	0.027	0.471
Selenium	rs921943	5	79,020,653	T	C	0.29	119	0.250	0.020	1.90E−39	0.069	0.029	0.019
Selenium	rs567754	5	79,120,593	C	T	0.67	67	0.170	0.020	8.40E−20	0.056	0.028	0.048
Selenium	rs3797535	5	79,004,574	T	C	0.10	36	0.210	0.040	2.10E−15	−0.011	0.049	0.816
Selenium	rs11951068	5	79,008,491	A	G	0.06	31	0.210	0.040	1.90E−11	0.076	0.051	0.137
Selenium	rs705415	5	78,996,137	C	T	0.88	39	0.230	0.040	4.60E−10	−0.008	0.042	0.841
Selenium	rs6586282	21	43,058,387	C	T	0.85	21	0.120	0.030	4.00E−09	−0.046	0.035	0.194
Selenium	rs1789953	21	43,062,826	T	C	0.16	17	0.120	0.030	3.40E−08	0.041	0.040	0.301
Iron	rs1800562	6	26,093,141	A	G	0.07	696	0.328	0.016	2.70E−97	−0.054	0.049	0.268
Iron	rs1799945	6	26,091,179	C	G	0.15	450	−0.189	0.010	1.10E−81	0.069	0.038	0.067
Iron	rs855791	22	37,462,936	A	G	0.55	807	−0.181	0.007	1.30E−139	0.028	0.027	0.299
Iron	rs8177240	3	133,477,701	T	G	0.67	95	−0.066	0.007	6.70E−20	0.005	0.028	0.860
Iron	rs7385804	7	100,235,970	A	C	0.62	95	0.064	0.007	1.40E−18	−0.003	0.028	0.918
Zinc	rs2120019	15	75,334,184	T	C	0.79	75	0.287	0.033	1.60E−18	0.021	0.034	0.537
Zinc	rs1532423	8	86,268,313	A	G	0.37	47	0.178	0.026	6.40E−12	−0.006	0.027	0.811
Copper	rs1175550	1	3,691,528	G	A	0.22	38	0.198	0.032	5.00E−10	0.003	0.032	0.922
Copper	rs2769264	1	151,344,741	G	T	0.16	85	0.313	0.034	2.60E−20	0.006	0.034	0.866

SNP, single-nucleotide polymorphism; Chr, chromosome; Pos, position; EA, effect allele; OA, other allele; EAF, frequency of effect allele; F, F-statistics; SE, standard error; and CAD, coronary artery disease.

**Figure 2:**
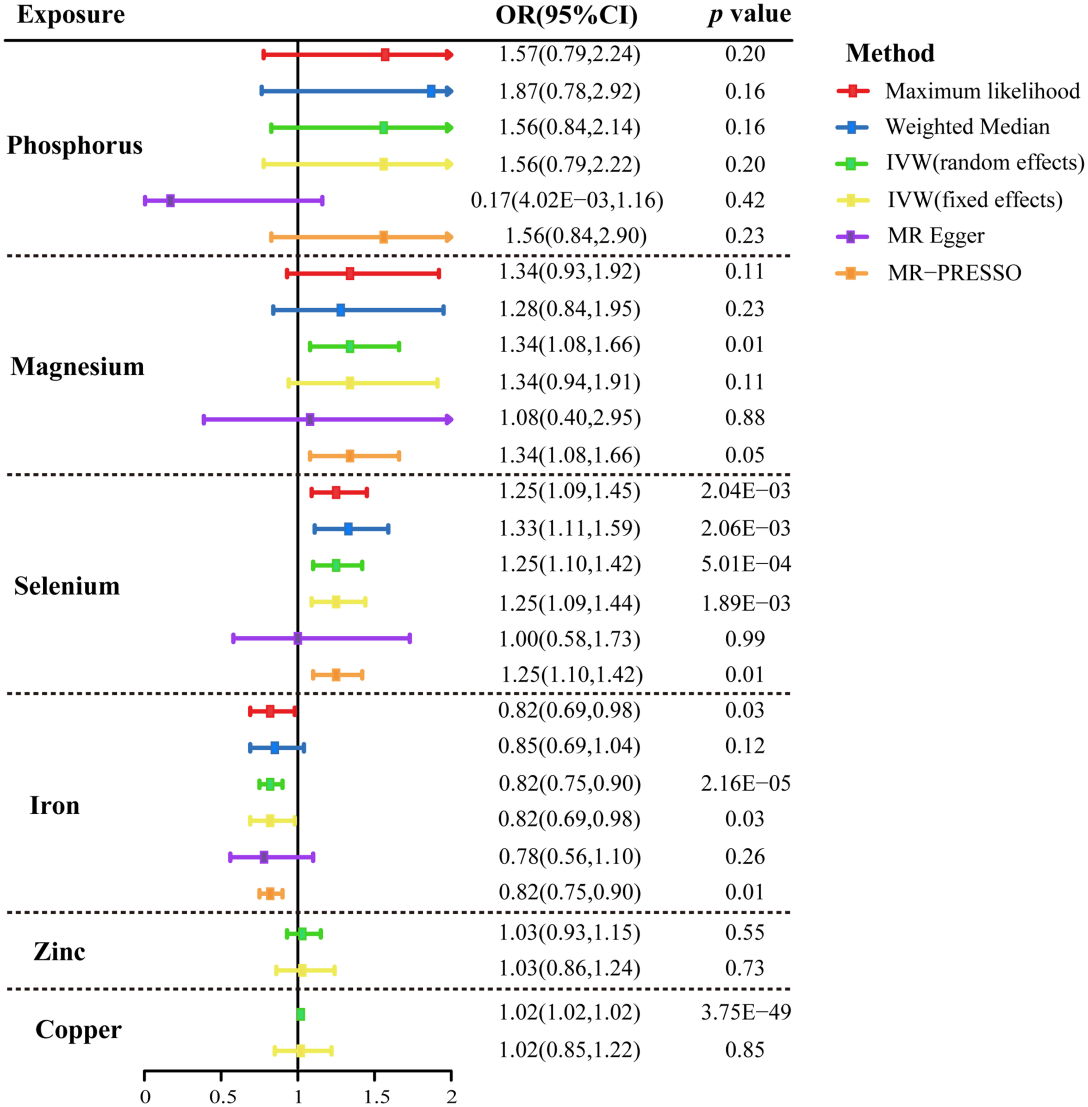
Mendelian randomization association of genetically predicted serum micronutrients levels with coronary artery disease in patients with diabetes using different statistical models. OR, odds ratio; CI, confidence interval; IVW, inverse-variance weighted method; MR, Mendelian randomization; and MR-PRESSO, MR-pleiotropy residual sum and outlier.

The primary findings of MR studies of genetically predicted circulation concentrations of micronutrients with the risk of CAD in individuals with diabetes were displayed in Figure 2. The random-effects IVW results indicated that genetically predisposition to one standard deviation increase in concentrations of serum copper, selenium, and magnesium was linked to 2% (odds ratio [OR], 1.02; 95% CI, 1.02–1.02 *p* = 3.75 × 10^−49^), 25% (OR, 1.25; 95% CI, 1.10–1.42; *p* = 5.01 × 10^−4^), and 41% (OR, 1.41; 95% CI, 1.14–1.73; *p* = 1.25 × 10^−3^) higher risk of CAD in diabetic patients, respectively (Figure 2). An 18% (OR, 0.82; 95% CI, 0.75–0.90, *p* = 2.16 × 10^−5^) reduced risk of CAD was observed in patients with diabetes when the genetically predicted serum iron content increase by one standard deviation (Figure 2). There was minimal proof that circulating concentrations of phosphorus and zinc were associated with the risk of CAD in patients with diabetes (Figure 2). The scatter plots also visually depicted the associations between micronutrients and CAD in diabetic patients (Supplementary Figures 1–6).

The association patterns of phosphorus, selenium, and iron based on sensitivity analyses were consistent with the IVW MR analyses, but not magnesium (Figure 2). In addition, stable correlations were found in the MR-PRESSO analysis of serum phosphorus (OR, 1.56; 95% CI, 0.84–2.90; *p* = 0.23), serum magnesium (OR, 1.34; 95% CI, 1.08–1.66; *p* = 0.05), serum selenium (OR, 1.25; 95% CI, 1.10–1.42; *p* = 0.01), and serum iron (OR, 0.82; 95% CI, 0.75–0.90; *p* = 0.01) with no outliers were revealed (Figure 2). Between the estimates of chosen SNPs, no evidence of heterogeneity for the relationships between micronutrients and CAD in diabetic patients was observed, and neither the MR-Egger intercept test nor the Cochrane’s *Q* test indicated any possible directional pleiotropy (all *p* > 0.05; Table 2). Leave-one-out analyses suggested that no single SNP significantly influenced the effect of serum micronutrient levels on CAD in diabetic patients (Supplementary Figures 7–10).

**Table 2:** Heterogeneity and pleiotropy tests for the associations of micronutrients with coronary artery disease in patients with diabetes.

Micronutrients	Q value	*p*_Q_	MR-Egger intercept	*p*_intercept_
Phosphorus	3.26	0.516	0.100	0.324
Magnesium	1.48	0.830	0.016	0.691
Selenium	4.78	0.572	0.044	0.454
Iron	1.03	0.905	0.009	0.761
Zinc	0.32	0.570	NA	NA
Copper	1.76E-04	0.989	NA	NA

MR, Mendelian randomization; NA, not available.

## Discussion

In this comprehensive MR analysis, genetic data from the largest published GWAS were leveraged to evaluate the relationship between genetic susceptibility to six micronutrients and the risk of CAD in diabetic patients. We provided consistent evidence that circulating selenium concentrations were genetically expected to be related with a higher risk of CAD, whereas iron concentrations were associated with a lower risk of CAD in patients with diabetes. The association pattern remained consistent when repeated in the majority of supplementary analyses. However, there was limited evidence to link the risk of CAD in diabetic patients with circulating levels of magnesium, phosphorus, zinc, and copper.

### Selenium and CAD in patients with diabetes

According to the previous observational studies and RCTs, the association between selenium and CAD in patients with diabetes was inconsistent (11, 27, 28). A prospective study involving 3,897 diabetes in the Dongfeng-Tongji cohort suggested an inverse association between plasma levels of selenium and risk of cardiovascular diseases (CVDs) in patients with diabetes (29). Selenium supplementation was not sufficient however, to reduce CAD mortality, according to the findings from a meta-analysis that included 16 RCTs (30). Additionally, previous observational studies have reported no difference in circulating selenium concentrations between diabetic patients with and without CAD (27). A positive association of selenium with diabetes was found in previous observational studies (31–33), several RCTs (34–36), and a MR study (37). Numerous *in vitro* and animal investigations have revealed the mechanism for how selenium increases the risk of diabetes and CAD. As a member of the glutathione peroxidase (GPx) family, selenium serves as the center of redox (38). Transgenic animal models have found increased GPx1 expression interferes with insulin signaling by removing hydrogen peroxide, leading to the development of insulin resistance, hyperglycemia, and obesity (39, 40). Selenoprotein P (SelP), a selenium-supply protein, is hypothesized to raise the risk of diabetes by promoting insulin resistance and dysregulating glucose metabolism (41). In addition, Selk, a selenoprotein of the endoplasmic reticulum membrane, contribute to foam cell formation and atherogenesis by stabilizing expression of CD36 in macrophages during inflammation (42, 43). According to the results of our MR investigation, selenium may be associated in a directionally consistent manner with CAD in patients with diabetes. Given that diabetes is a known risk factor for CAD, this conclusion might seem intuitive. However, considering the majority of the individuals covered with this research were of European origin, the generalizability of our findings to other groups needs to be further investigated. The inverse association between levels of selenium and CVDs risk in Asian diabetic may due to the difference of dietary structure, lifestyle and genetic predisposition.

### Iron and CAD in patients with diabetes

An inverse association between iron concentration and CAD in diabetic patients was observed in our MR analysis. A two-sample MR approach examining serum iron status for CAD risk in the general population revealed that serum iron concentration was linked to a lower chance of developing CAD (OR, 0.94; 95%CI, 0.88–1.00; *p* = 0.039), which is consistent with our findings in the diabetic population (44). In addition, a recent two-sample MR study based on the data from United Kingdom Biobank discovered that high levels of iron status were protective against coronary atherosclerosis in the male population (45). Furthermore, a meta-analysis of prospective studies involving 156,427 participants showed a negative association between serum iron and risk of coronary heart disease after excluding the study by Morrisson et al. (risk ratio [RR], 0.80; 95%CI, 0.73–0.87) (46). Numerous observational studies have also demonstrated the protective effect of iron on CAD in diabetic individuals (47, 48). An inverse correlation between iron reserves and cardiovascular disease in patients with diabetes was reported by a cross-sectional and prospective observational study encompassing 38,671 people and 821 diabetes patients (OR, 0.81; 95%CI, 0.68–0.96; *p* = 0.018) (48). Similarly, the results of an observational study including 424 consecutive men with type 2 diabetes mellitus showed high ferritin levels may reduce cardiovascular risk in men with diabetes (49). Several plausible mechanisms have been hypothesized to elucidate the protective effect of high iron load on CAD. For instance, an animal study found that a high-iron diet attenuates atherosclerosis in mice lacking apolipoprotein E (50). Similar, another recent animal study suggested that iron overload could diminish atherosclerosis in apolipoprotein E knockout mice by interfering with hepatic CD36 and fatty acid binding proteins-mediated fatty acid uptake and transport (51). Furthermore, it has been proven that ferritin, a natural antioxidant, may reduce the risk of CAD in patients with diabetes by compensating for chronic systemic inflammation in diabetes (52, 53).

### Other micronutrients and CAD in patients with diabetes

In the current MR study, we observed a nominally significant association between genetically predicted concentrations of magnesium and the risk of CAD in patients with diabetes, but the other four statistical models were not statistically significant. As a result, we preclude the presence of a stable causal association between serum magnesium concentration and outcome. We also observed a significant correlation between genetically predicted concentrations of copper and the risk of CAD in patients with diabetes in the main analysis; however, because there are only two genetic instruments for copper, we are unable to perform sensitivity analysis to assess the stability of the results. Meanwhile, the results of IVW (fixed effects) suggested no causal connection between copper and CAD in patients with diabetes. Thus, we are unable to tell whether there is a possible causal relationship between copper and the outcome. The results of the current MR study showed that little evidence approved the causal effects of genetically predicted concentrations of phosphorus and zinc on CAD risk in diabetic patients. There is a scarcity of observational epidemiology research on these micronutrient concentrations and the incidence of CAD in diabetic patients, and the results from the few available observational studies of the general population are inconclusive (12, 54–57). Thus, our results from the current MR study may imply that serum phosphorus and zinc levels should not be regarded as independent risk factors for CAD in patients with diabetes.

### Strengths and limitations

The design of MR study, which avoids biases frequently seen in standard observational studies and provides the non-biased causal connection between exposure and outcome, is the main merit (58). Besides, our MR study uses summary-level data from the large genetic consortium to date, which allows us to more accurately formulate our study hypothesis. Meanwhile, the statistical power in the current investigation is ensured by the estimated effects (*F*-statistics) of each instrumental variable exceeding the threshold. Moreover, sensitivity analyses based on multiple statistical models combined with leave-one-out analyses were employed to detect the stability of the main results, which offered additional reliable evidence.

It is crucial to acknowledge several potential limitations when interpreting our results. First, although MR-PRESSO analysis and MR-Egger intercept tests did not reveal any evidence of pleiotropy that might have influenced our results, potential horizontal pleiotropy cannot be completely excluded. Second, the current study was based on summary-level data and lacked subgroup-specific analyses, as there are no corresponding sex- or age-specific data sets in the consortium. Third, the majority of the individuals in our MR research were of European origin, which may restrict the generalizability of the primary findings to other groups. Therefore, the corresponding results should be cautious to make the conclusion.

## Conclusion

The current study provides genetic evidence for the possible causal effects of increased selenium and decreased iron levels on the increased risk of CAD in patients with diabetes. Diet, supplements, or other methods to modify circulating selenium and iron concentrations may be effective strategies to prevent CAD in patients with diabetes.

## Data availability statement

The datasets presented in this study can be found in online repositories. The names of the repository/repositories and accession number(s) can be found in the article/Supplementary material.

## Ethics statement

Ethical review and approval was not required for the study on human participants in accordance with the local legislation and institutional requirements. The patients/participants provided their written informed consent to participate in this study.

## Author contributions

MT, NH, TH, and HC designed the study and wrote the analysis plan. NH and TH undertook analyses. MT and TH wrote the first draft of the manuscript with critical revisions from NH, JY, and HC. MT, TH, JY, HC, and NH interpreted the results in the study and gave final approval of the version to be published. All authors contributed to the article and approved the submitted version.

## Funding

This work was supported by grants from the Key Laboratory of Precision Medicine for Atherosclerotic Diseases of Zhejiang Province, China (Grant No. 2022E10026), National Natural Science Foundation of China (82200489), the Major Project of Science and Technology Innovation 2025 in Ningbo, China (Grant No. 2021Z134), the Key research and development project of Zhejiang Province, China (Grant No. 2021C03096), and Public Science and Technology Projects of Ningbo (202002N3175).

## Conflict of interest

The authors declare that the research was conducted in the absence of any commercial or financial relationships that could be construed as a potential conflict of interest.

## Publisher’s note

All claims expressed in this article are solely those of the authors and do not necessarily represent those of their affiliated organizations, or those of the publisher, the editors and the reviewers. Any product that may be evaluated in this article, or claim that may be made by its manufacturer, is not guaranteed or endorsed by the publisher.

## Footnotes

^1^ https://github.com/MRCIEU/TwoSampleMR

^2^ https://github.com/rondolab/MR-PRESSO
