# Supplementary material for: Increased selenium and decreased iron levels in relation to risk of coronary artery disease in patients with diabetes
Source: Front Nutr. 2023 May 18;10:1103330. doi: 10.3389/fnut.2023.1103330 (PMC10233138; doi:10.3389/fnut.2023.1103330)

Figure Legends

Supplemental figure 1. The scatter plot for the association between serum phosphorus and coronary artery disease in patients with diabetes.

Supplemental figure 2. The scatter plot for the association between serum magnesium and coronary artery disease in patients with diabetes.

Supplemental figure 3. The scatter plot for the association between serum selenium and coronary artery disease in patients with diabetes.

Supplemental figure 4. The scatter plot for the association between serum iron and coronary artery disease in patients with diabetes.

Supplemental figure 5. The scatter plot for the association between serum zinc and coronary artery disease in patients with diabetes.

Supplemental figure 6. The scatter plot for the association between serum copper and coronary artery disease in patients with diabetes.

Supplemental figure 7. The leave-one-out analysis for the association between serum phosphorus and coronary artery disease in patients with diabetes.

Supplemental figure 8. The leave-one-out analysis for the association between serum magnesium and coronary artery disease in patients with diabetes.

Supplemental figure 9. The leave-one-out analysis for the association between serum selenium and coronary artery disease in patients with diabetes.

Supplemental figure 10. The leave-one-out analysis for the association between serum iron and coronary artery disease in patients with diabetes.

Supplemental figure 1.

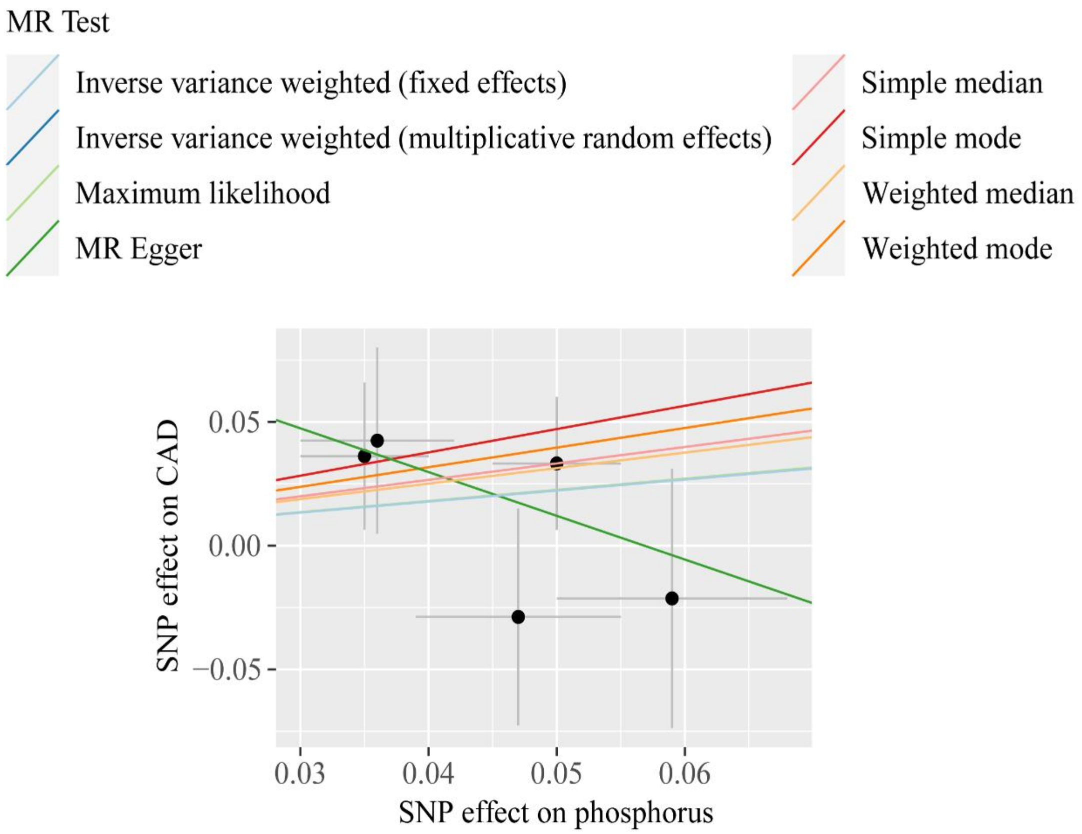

Supplemental figure 2.

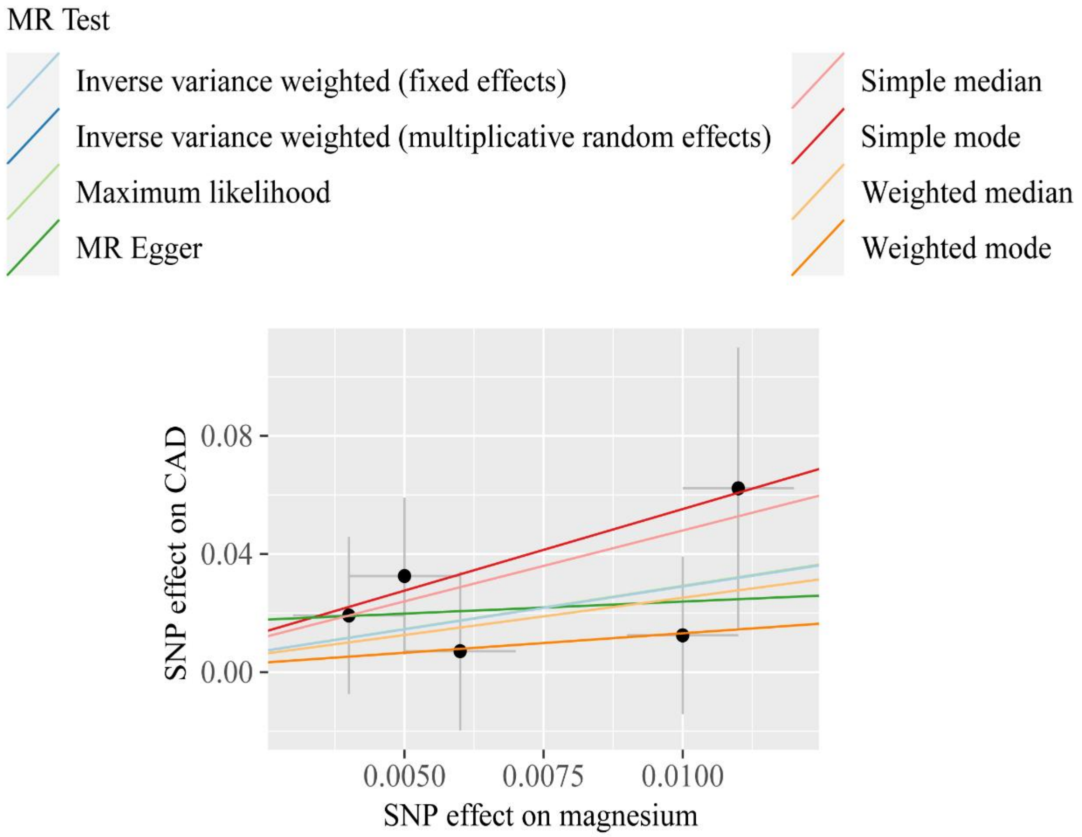

Supplemental figure 3.

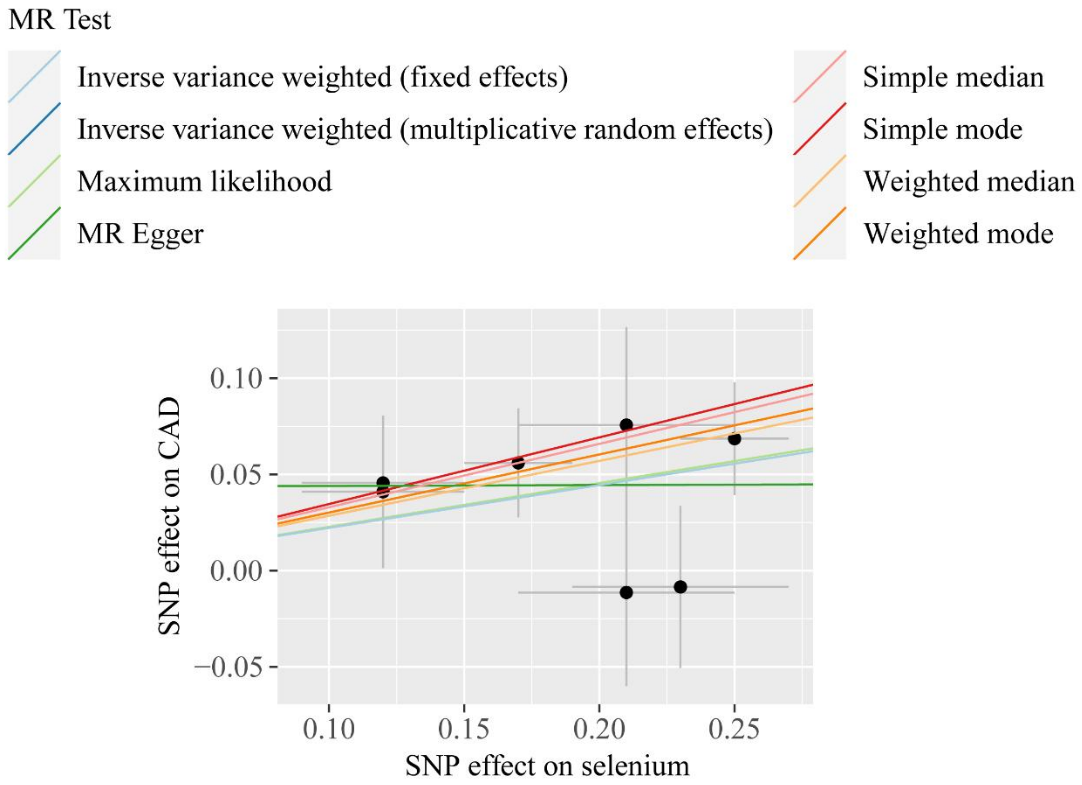

Supplemental figure 4.

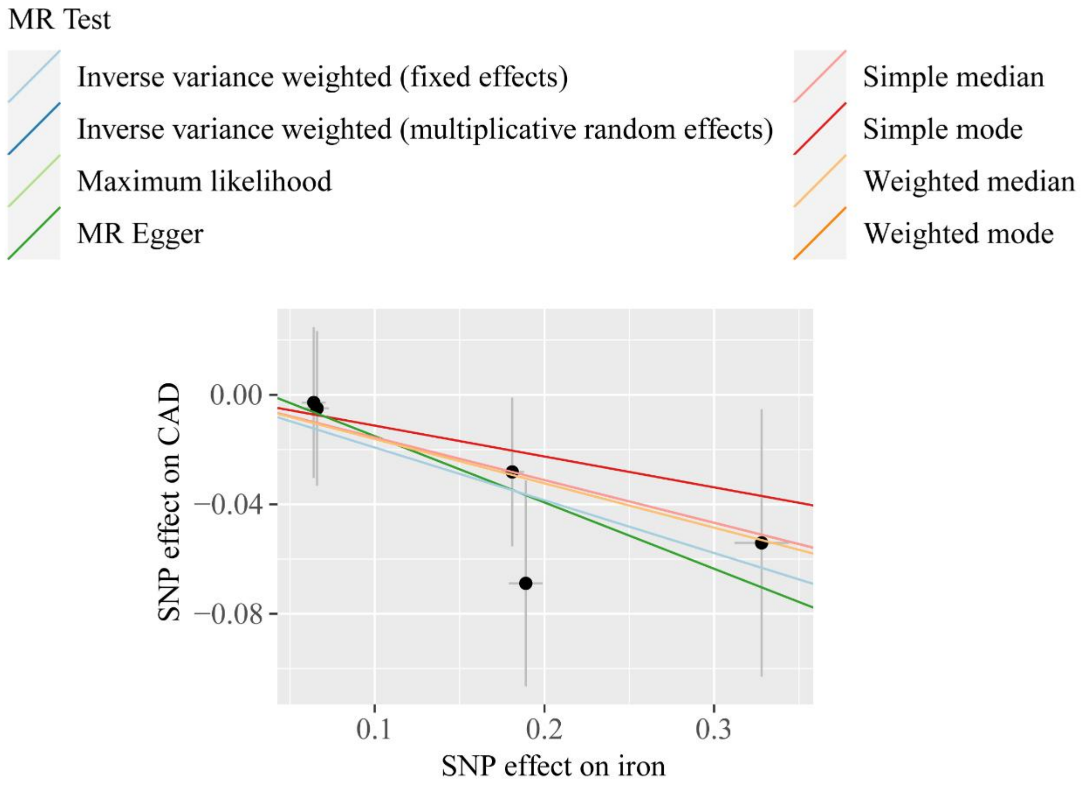

Supplemental figure 5.

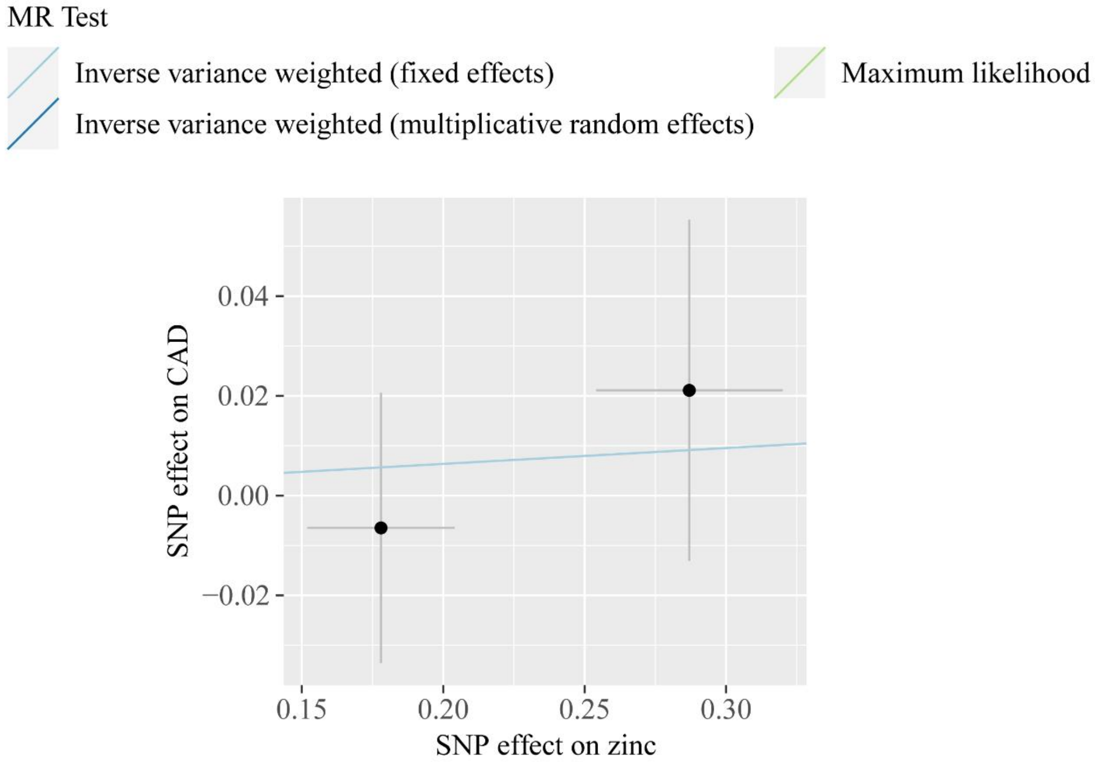

Supplemental figure 6.

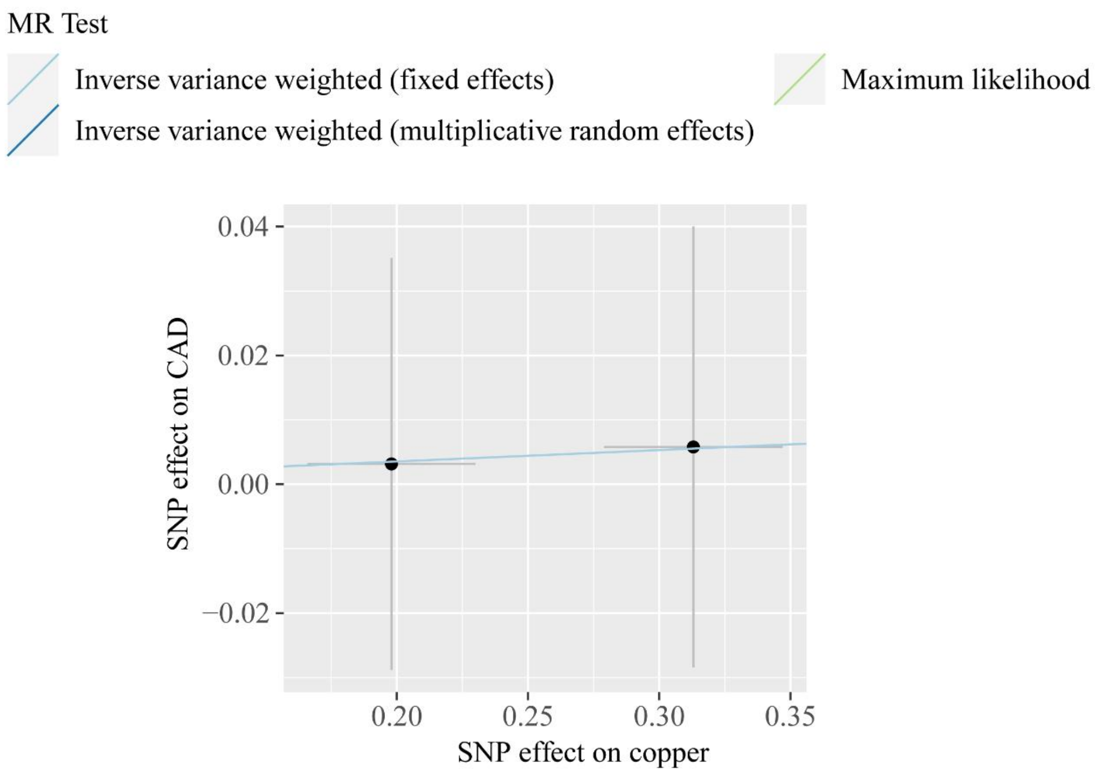

Supplemental figure 7.

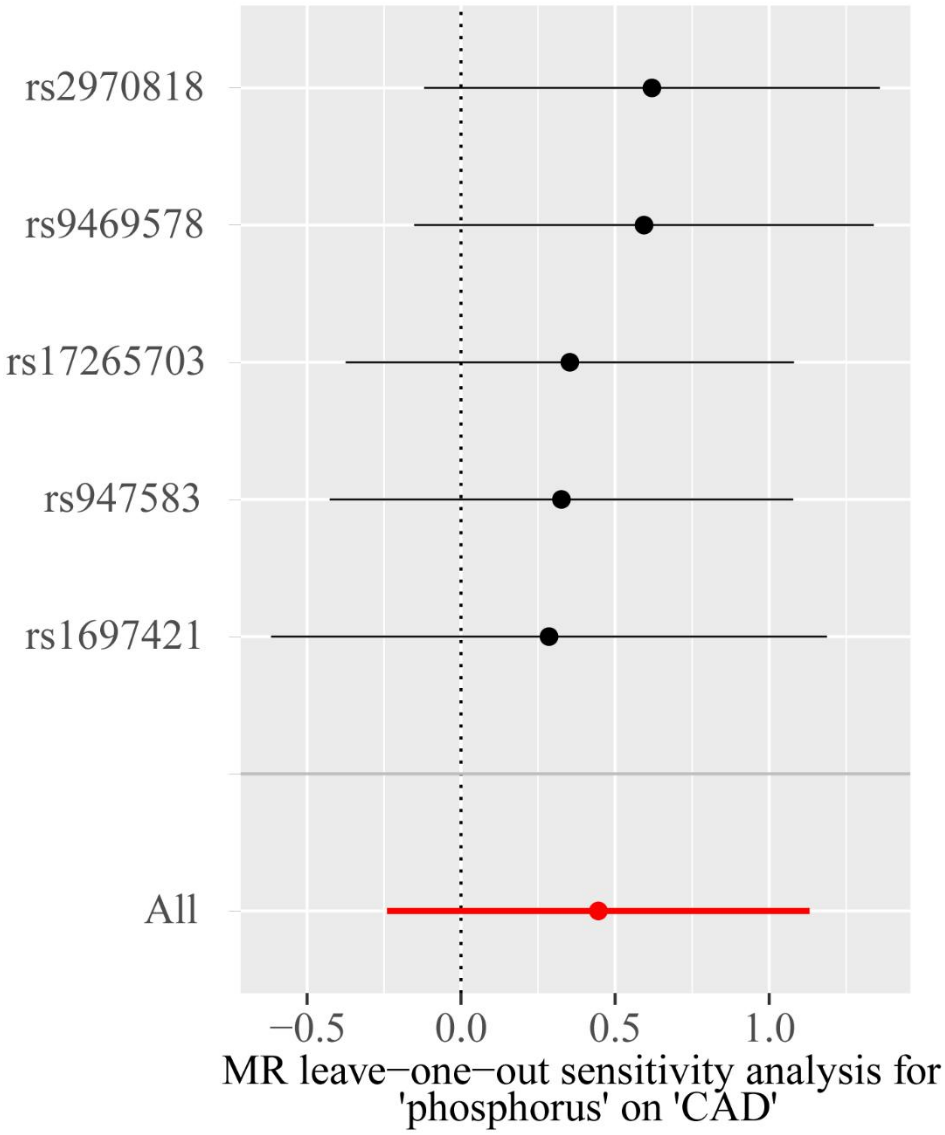

Supplemental figure 8.

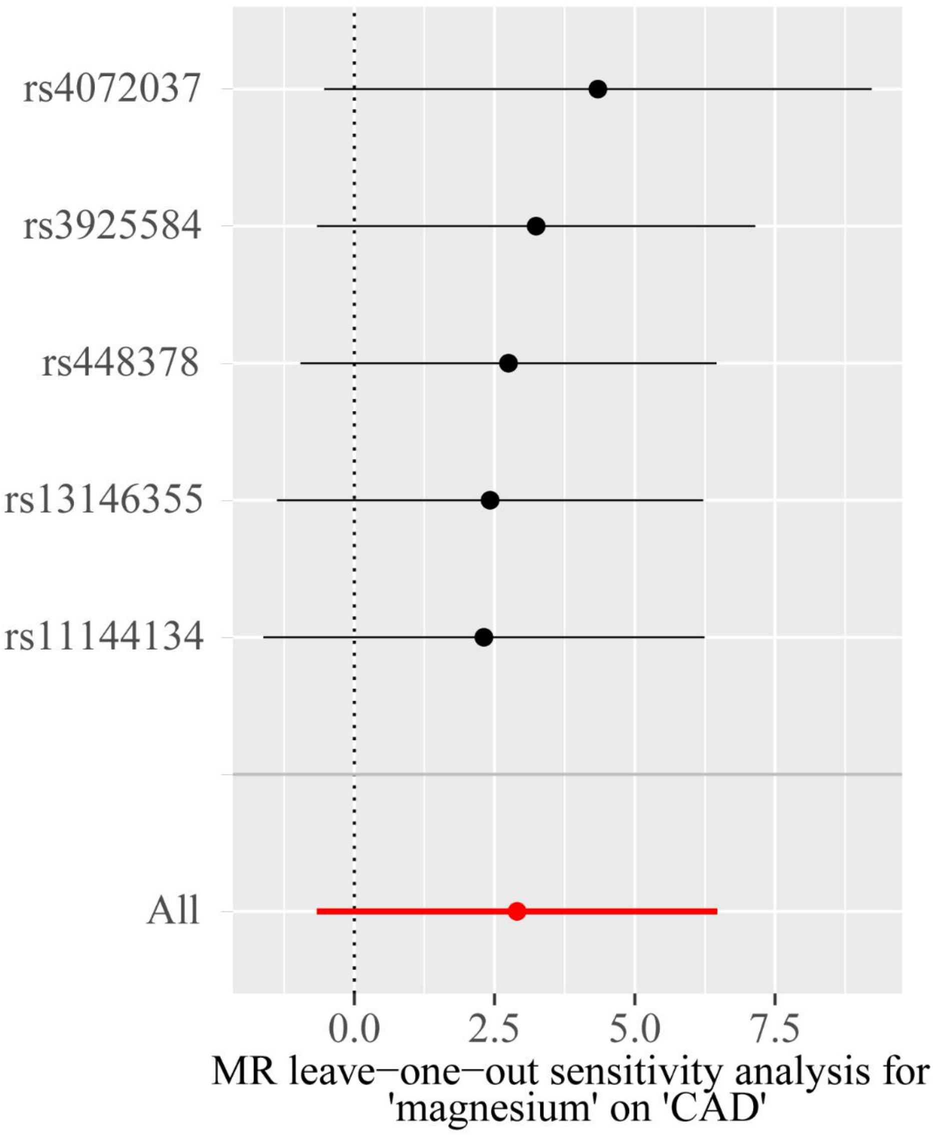

Supplemental figure 9.

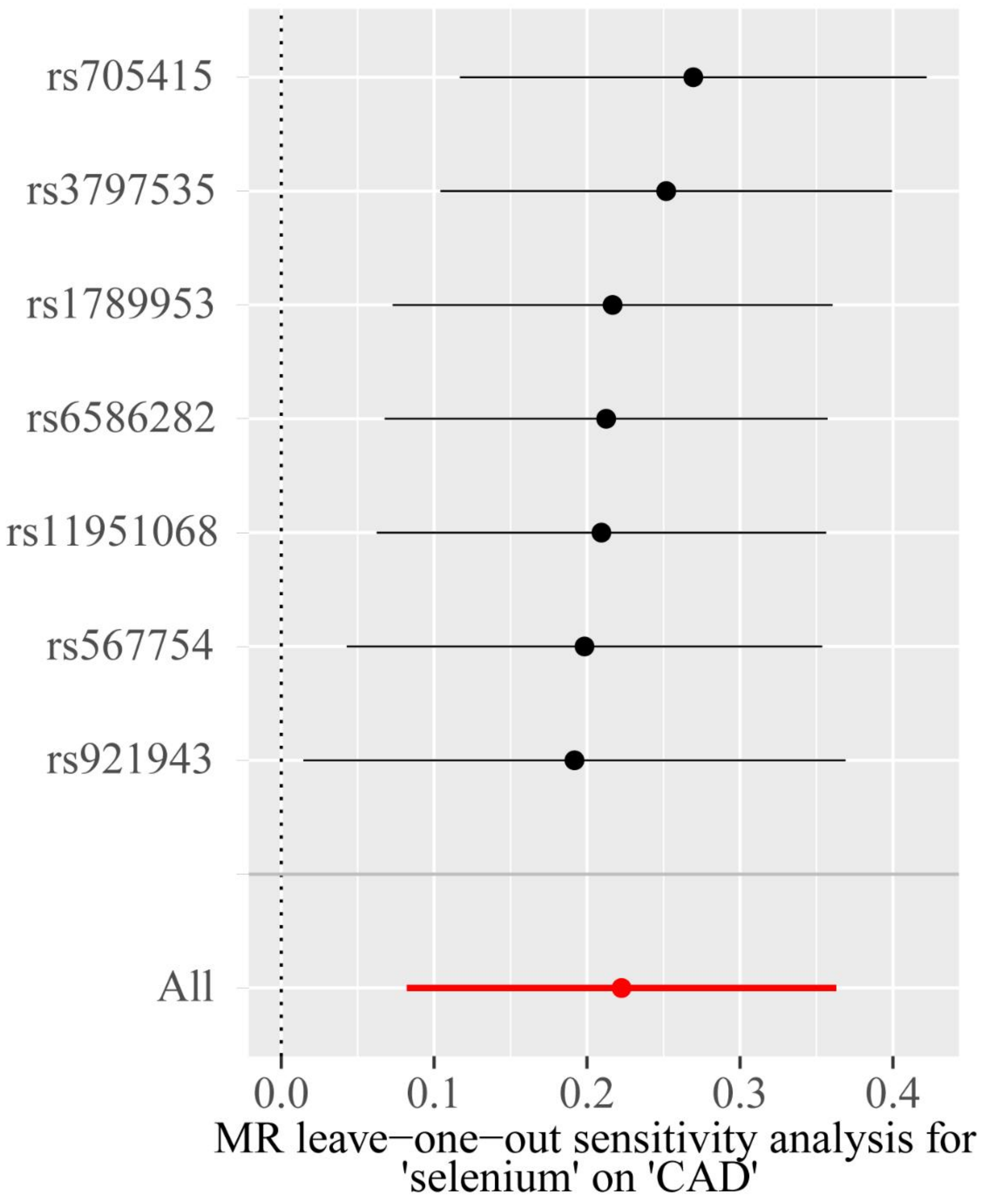

Supplemental figure 10.

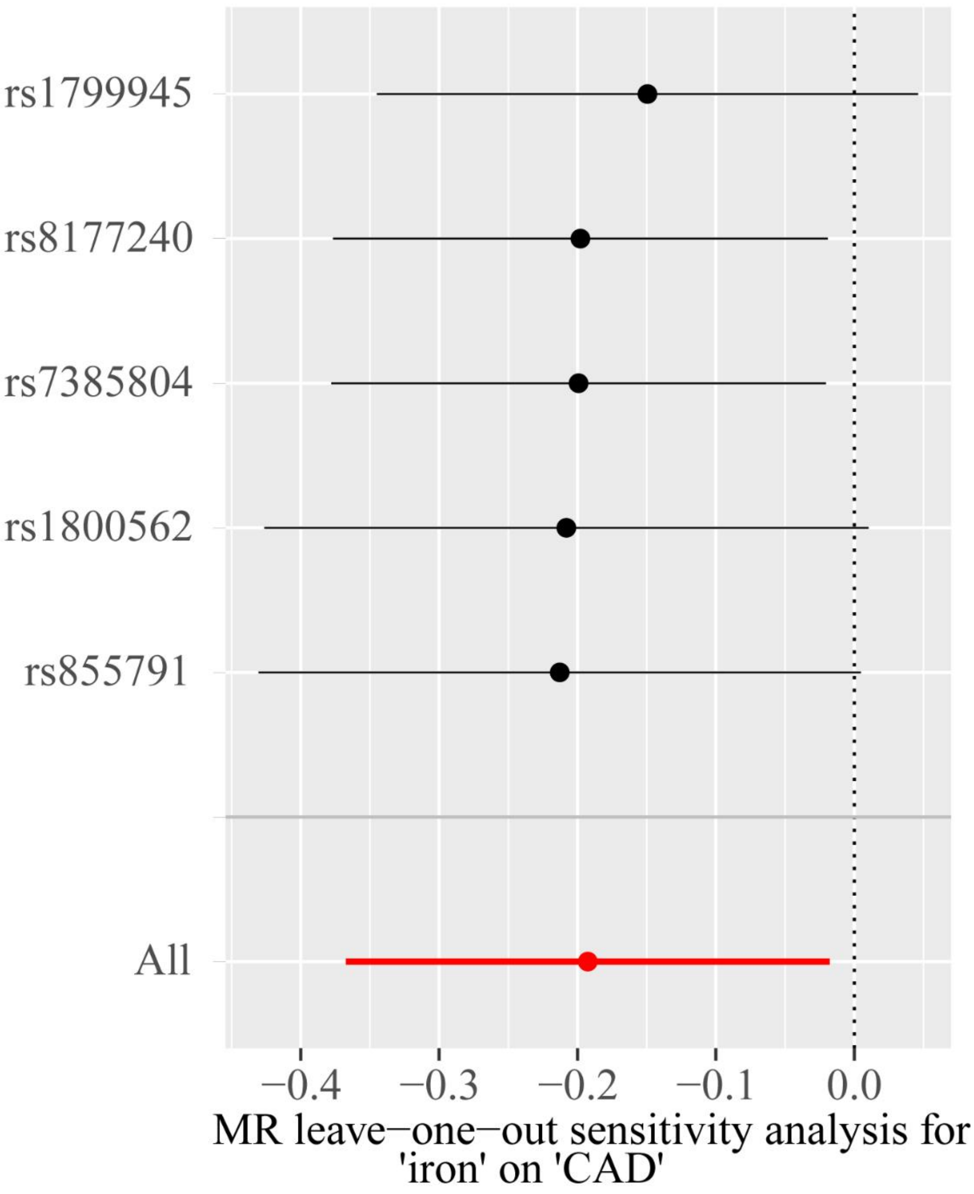

Supplement: Supplementary file 1 [file Data_Sheet_1.PDF]
